# Hemodynamic and Radiological Classification of Ovarian Veins System Insufficiency

**DOI:** 10.3390/jcm10040646

**Published:** 2021-02-08

**Authors:** Cezary Szary, Justyna Wilczko, Michal Zawadzki, Tomasz Grzela

**Affiliations:** 1 Clinic of Phlebology, Wawelska 5, 02-034 Warsaw, Poland; justyna.wilczko@klinikaflebologii.pl (J.W.); michal.zawadzki@klinikaflebologii.pl (M.Z.); tomasz.grzela@klinikaflebologii.pl (T.G.); 2 Diagnostic Imaging Center MRI&CT, Center of Sports Medicine, Wawelska 5, 02-034 Warsaw, Poland; 3 Department of Radiology, Centre of Postgraduate Medical Education, 01-813 Warsaw, Poland; 4 Department of Histology and Embryology, Medical University of Warsaw, 02-022 Warsaw, Poland

**Keywords:** ovarian veins, pelvic veins insufficiency, color-duplex ultrasound, venography

## Abstract

Ovarian veins system insufficiency is one of the most common reasons for pelvic venous insufficiency (PVI). PVI is a hemodynamic phenomenon responsible for the occurrence of venous insufficiency of the lower extremities and recurrent varicose veins in nulliparous and parous women, as well as for a set of symptoms described as pelvic congestion syndrome (PCS). In the years 2017–2019, 535 patients admitted to our center with symptoms of venous insufficiency of the lower extremities, underwent complete ultrasound diagnostics (color-duplex ultrasound) of the venous system of the abdomen, pelvis and lower limbs, as well as extended imaging diagnostics using computed tomography (CT) or magnetic resonance (MR) venography. On the basis of the obtained results, the authors proposed a 4-grade hemodynamic and radiological classification (grades I-IV) defining the stratification of ovarian veins insufficiency. Using the above mentioned classification approx. 32% patients were identified as Grade I and I/II, approximately 35% revealed morphological and hemodynamic changes corresponding to Grade II and II/III, approximately 25% were classified as Grade III, whereas the remaining 8% were assessed as Grade IV. The described classification allows for the grading of ovarian veins insufficiency based on transparent radiological criteria, making it easy to use in everyday clinical practice. According to the authors, the proposed classification could facilitate communication between diagnostic physicians, specialists dealing with the treatment of venous insufficiency and gynecologists, who admit patients with symptoms suggesting venous insufficiency of the pelvis.

## 1. Introduction

**Figure 1:**
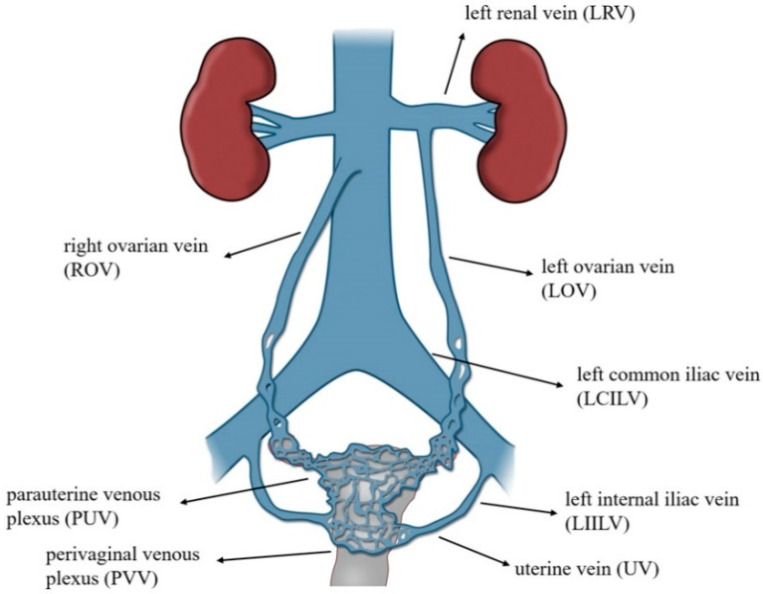
Illustration of abdominal and pelvic venous system in women.

Pelvic venous insufficiency (PVI) is a very common hemodynamic phenomenon (estimated to be present in 20–43% of the population in women) [1,2,3], most often resulting from the reversal of venous blood flow, which occurs in the mechanism of outflow disorders, or excessive dilatation of the main venous axes (VAX) located within the abdomen and pelvis. The abovementioned pathophysiological phenomenon results in overloading of the capacitive venous vessels in the pelvis, most often the para-uterine and peri-vaginal venous plexuses (Figure 1), thus generating so-called venous leaks from the pelvis and vulvo-perineal varicosities.

The phenomenon of pelvic venous insufficiency is most frequently caused by the hemodynamic dysfunction of the ovarian veins [4,5,6,7], leading to the retention of venous blood in the para-uterine venous plexuses, which through anatomical anastomoses, including uterine veins, overloads the internal iliac veins. This phenomenon is particularly evident in multiparous women who develop chronic insufficiency of both ovarian veins after delivery and widening of the branches of the internal iliac veins.

In the light of our recent studies [8], growing evidence indicates that PVI resulting from the incompetence of the ovarian veins is responsible in part for primary venous insufficiency of the lower extremities, as well as for the formation of recurrent varicose veins. As we know from the latest literature of patients with recurrent varices of the lower extremities, PVI is found in over half of studied women [7,8,9,10,11,12,13].

Pelvic venous insufficiency can cause a number of clinical symptoms. Some of them, limited to the pelvis and genital areas (most often perineum and vulva), are defined as pelvic congestion syndrome (PCS). Typical PCS is characterized by gravitational pelvic pain or discomfort that is worse in the upright and sitting position and is associated with pelvic and vulvo-pudendal varicosities as well as symptoms of dyspareunia and postcoital pain [9,10].

The first attempts to systematize the diagnostic approach to pelvic venous insufficiency began in the 1990s. For many years, the gold standard of imaging in PVI has been classical retrograde phlebography, performed by percutaneous access with direct catheterization and contrasting of potentially failing vessels, such as ovarian veins or internal iliac veins [14,15,16]. The development of ultrasonography, and especially the improvement of duplex ultrasound techniques, enabled the use of transvaginal ultrasound (TVU), and later of transabdominal ultrasound (TAU) in the diagnosis of PVI. Numerous attempts were also made to standardize the examination of the venous system of the pelvis and the abdominal cavity using duplex ultrasound [16,17,18,19,20].

The following years saw the development and increase in the frequency of angiographic examinations in computed tomography and magnetic resonance imaging. [4,21,22,23]. The first attempts were also made to standardize this type of research and to create the first classifications describing the phenomenon of pelvic venous insufficiency [4,21,22,23]. In the newest international consensuses [24,25] the authors briefly present 4-grades (GI-GIV) of pelvic venous reflux criteria for cross-sectional imaging diagnosis of PVI.

However, to date, none of the existing classifications has gained universal approval [25]. Taking the above into account, the authors attempted to create a simple hemodynamic–radiological classification that would stratify the grade of ovarian veins system insufficiency. For this purpose, a retrospective analysis of the results of various imaging examinations was performed, including venous color-duplex ultrasound (CDU), intravascular ultrasound (IVUS), magnetic resonance venography (MRV), computed tomographic venography (CTV) and digital phlebography results collected in the database of our clinic.

## 2. Methods and Diagnostic Approach

The retrospective analysis covered data collected in the database of our clinic in the years 2017–2019. The data originated from 535 records (C1-C4 in CEAP classification) corresponding to women subjected to routine diagnostics and treatment procedures for venous insufficiency, according to a standardized protocol. The study was formally approved by the Local Ethics Committee at the Medical University of Warsaw (decision No. AKBE/181/2020).

The initial diagnostic approach for a patient with suspected pelvic venous insufficiency was based on transabdominal color-duplex ultrasound of the venous system (TAU). A convex probe with a frequency of 6 to 8 MHz was used. Patients were prepared for the examination with 12 h fasting after taking degassing agents, in order to optimize vein evaluation. The TAU examination is performed in a lying or half-sitting position, and in doubtful cases also in a sitting position. The examination with CDU targets the morphology of the venous system, the interrelationships of the main venous axes (IVC-inferior vena cava, LRV-left renal vein, RRV-right renal vein, LOV-left ovarian vein, ROV-right ovarian vein, CILV-common iliac veins, EILV-external iliac veins and IILV-internal iliac veins) and the venous flow in free-breathing, during prolonged inspiration and breathing tests as well as during the Valsalva maneuver. Initial TAU assessment is correlated with percutaneous CDU of the veins of the genital area and lower extremities and the symptoms reported by the patient, often typical of pelvic congestion syndrome (PCS). This allows the addressing of proper further selected imaging tests to complement diagnostics when needed. This extended diagnostics also aimed at excluding secondary causes of venous insufficiency (e.g., neoplasms or venous entrapment syndrome caused by bone compression); more importantly, additional diagnostic imaging examinations contribute to qualify the patient for further endo-venous treatment (e.g., venous embolization, venous angioplasty, or venous stenting).

CT and MR venography imaging (CTV and MRV) was routinely used in the extended diagnostics. In both cases, a contrast agent was administered via a pump with a 3.5–4 mL/s flow. The scope of the study included the patient’s body from the base of the heart (IVC flow into the right atrium of the heart must be visualized) to the upper third of the thighs.

In the case of CTV examinations performed on the 64-row and 128-slice system (Incisive CT scanner by Philips), two venous phases were performed: early (approximately 50 s after reaching the peak saturation with the contrast agent in the abdominal aorta) and late (approximately after 120 s).

In the case of MRV examinations performed on the 3 Tesla MRI system (Ingenia scanner by Philips), the following scans were performed: non-contrast morphological sequences (T2, FatSat T2, BTFE–balanced turbo field echo), post-contrast dynamic sequences consisting of 2–6 phases of contrast as well as high-resolution contrasting of pelvis (mDIXON, one of the modified fat saturation techniques using alternative water/fat phase-encoding strategies) including the pelvis (just above the L4/L5 level), the perineal and the vulvar region.

All patients undergoing diagnostic imaging were assessed for the presence of anatomical variations and venous compression phenomena (e.g., nutcracker syndrome); moreover a standardized measurement of venous trunks was taken. The diameters of the ovarian axes were measured in the upper fourth and the lower third. The measurements of vessel diameters were always taken from wall to wall in a plane perpendicular to the vessel’s long axis. The dimensioning accuracy was up to 0.5 mm. When assessing vein hemodynamics, ovarian veins system insufficiency was defined as a prevalent reversed centrifugal flow during free-breathing or during Valsalva maneuver.

## 3. Results

### 3.1. Ovarian Veins Morphology

The data used for assessment concerned the records of 535 women from our database. The short clinical characteristics of the analyzed group are shown in Table 1.

**Figure 2:**
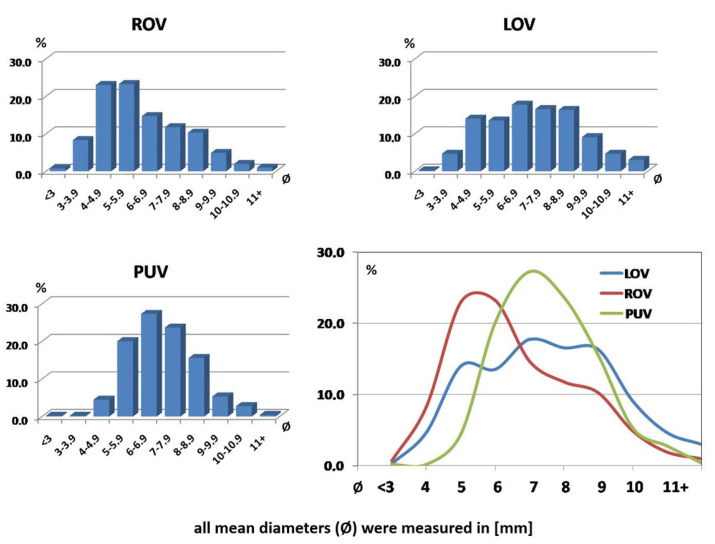
Main numerical-parametric relationships resulting from the analysis of 535 patients from our database, carried out in 2017–2019.

The TDU revealed the clinically relevant dilatation (Ø > 6 mm) of assessed veins, especially LOV and para-uterine veins, in approx. 67.5% women. The marked dilatation of ROV concerned approximately 33% patients. In all mentioned cases the vein dilatation was accompanied by reversed blood flow or stasis (especially in PUV). Noteworthy, all these findings were further confirmed in either CTV or MRV. The data concerning the distribution of ovarian and para-uterine veins diameter in assessed group are shown on Figure 2.

**Figure 3:**
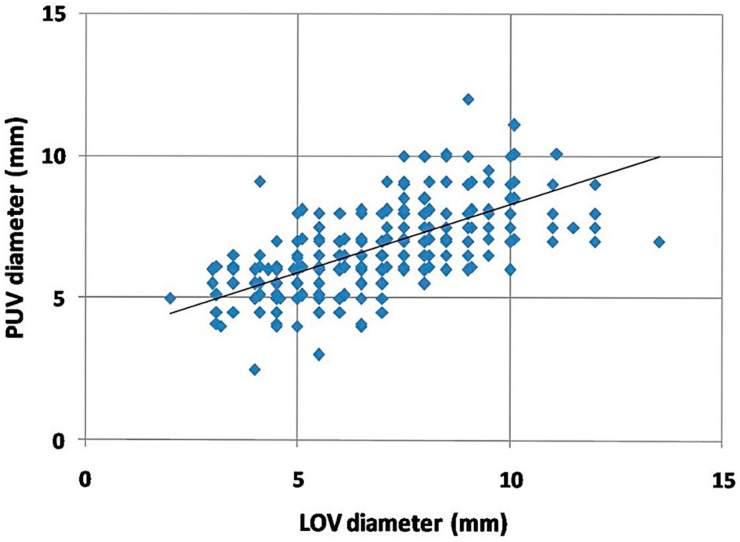
Correlation of the dilatation of the para-uterine venous plexuses (PUV) compared to the dilatation of the left ovarian vein (LOV) trunk.

The incompetence and significant dilatation of the ROV occurred later than in the left, and usually in this group of women only after two deliveries. There was also a correlation between the left ovarian vein dilatation and the dilatation of the para-uterine venous plexuses (Figure 3). Such a relationship may suggest that the gradual left-right overload model best reflects the most common mechanism of PVI in women.

### 3.2. Anatomical Variations

As much as 67.5% of women diagnosed in the study due to symptomatic venous insufficiency of the lower extremities showed ovarian vein enlargement and reflux (morphological criterion for the incompetence of LOV or ROV was Ø > 6 mm). A total of 37.4% of patients demonstrated the existence of various anatomical factors and developmental variations of the venous system (LRV anomalies 10.1%, LOV anomalies 8.8%, ROV anomalies 4.5%, ILV anomalies 10.6%, IVC anomalies 2.2%), significantly influencing the functioning of the left (Table 2) and right ovarian axis (Table 3). Both tables summarize the encountered findings.

## 4. Proposal for a Hemodynamic and Radiological Classification of Ovarian Veins Insufficiency

### 4.1. Assumptions of the Ultrasound and Radiological Classification of Ovarian Veins System Insufficiency

Based on the CDU, MRV and CTV findings a detailed morpho-hemodynamic classification of ovarian veins system insufficiency is proposed (Table 4). Four grades of veins insufficiency (I-IV) are proposed. There are also two intermediate grades marked with symbols I/II and II/III (Table 4).

**Figure 6:**
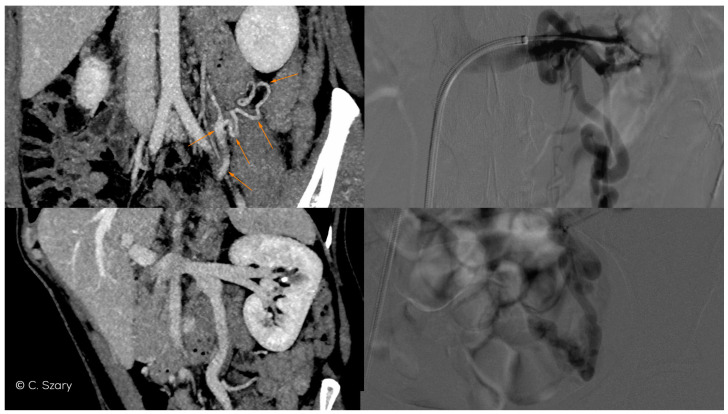
CTV and intra-interventional phlebographic images of nutcracker phenomenon (LRV compression by superior mesenteric artery, dilated venous collaterals from hilar veins of left kidney (orange arrows), insufficient LOV).

Anatomical variations and significant developmental variants may affect the female pelvic venous system even in teenagers, before any pregnancy takes place. These variations may increase intravenous blood pressure, with reversed flow and/or collateral circulation bypassing the vein obstruction or compression. Most often these changes concern LRV-LOV axis involving the ascending lumbar vein or internal iliac veins (Figure 6).

**Figure 7:**
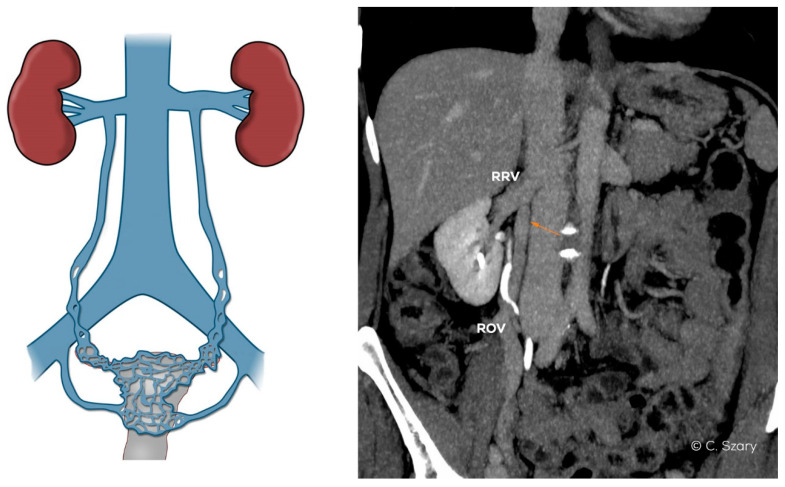
Atypical drainage of insufficient right ovarian vein (ROV) into right renal vein (RRV) on CTV image.

In the assessed material, it was found that the most common anatomical phenomena and variations, significantly affecting morphology and hemodynamics of the ovarian axes, are:

− narrow distal segment of the LRV (developmental variant);
− extrinsic compression of the LRV by the superior mesenteric artery (SMA)–typical of the nutcracker phenomenon;
− atypical drainage of the right ovarian vein into the trunk of the right renal vein (Figure 7).

The proposed grading of venous insufficiency of the ovarian venous system takes into account various factors, assessed both in ultrasound and imaging examinations, such as CTV and MRV, including:

− the mean diameter of ovarian veins (the upper and lower segments are always assessed in the late phase of contrast enhancement);
− the rate and order of the ovarian veins contrasting;
− the dynamics of contrast enhancement in ovarian and iliac veins, assessed in dynamic radiological sequences;
− the mean diameter of the internal iliac veins and their branches;
− maximum distension of the para-uterine venous plexuses on both sides;
− the presence of collateral venous circulation generated as a result of venous outflow disorders (most often in the LRV axis drainage);
− occurrence of venous anastomoses in the pelvis (the direction of flow can be assessed in the CDU, and often also in the dynamic contrast enhanced MRV examination).

Taking into account the above criteria and classification, a better defined qualification of patients for an appropriate endo-venous procedure is possible.

### 4.2. Grading of Ovarian Veins Insufficiency

The conducted analysis as well as the data from the literature show that the most common mechanism of pelvic venous insufficiency in women is the result of post-pregnancy dilatation of the left ovarian vein as well as flow abnormalities in the left renal-ovarian axis. LOV dilatation (and insufficiency) increases with each subsequent full-term pregnancy [6,26]. Persistent LOV incompetence most often occurs after the 2nd or 3rd delivery. The constant overload of the para-uterine venous plexuses leads to their distension with possible volumetric overload of the right ovarian vein system and outflow of the internal iliac veins (more frequently on the left side). Pelvic venous insufficiency caused by outflow obstruction through the inferior vena cava axis and the right ovarian vein axis incompetence is much less frequently observed.

It is likely that the described mechanism may be of particular importance in those patients who exhibit the anatomical variations described previously, but in whom hemodynamic disturbances are initially mild or moderate but worsen significantly during pregnancy.

The ovarian venous axes in the proposed classification are characterized by their maximum diameter (_Ø_LOV and _Ø_ROV) and venous flow competence (inc = incompetence; incLOV, incROV). Based on the measurements carried out in a healthy population, it was assumed that the LOV trunk diameter (measured in the supine position) should be in the range of approx. 3.5–5.5 mm, and in the case of ROV trunk, usually of 3–5 mm. Similar observations come from studies in which patients with PVI were imaged [17,27].

Venous insufficiency of the ovarian axes can be assessed by determining the dominant direction of blood flow in the CDU examination or by the rate and sequence of contrast enhancement in the dynamic MRV examination. It can also be approximated by analyzing the rate of contrast filling of venous trunks in the CTV examination. This classification also takes into account the assessment of the degree of dilatation of the periovarian and para-uterine venous plexuses defined commonly as the para-uterine veins (lPUV, rPUV). Under normal conditions, the diameter of the para-uterine venous plexuses should not exceed 4–5 mm [22,28].

Another parameter included in the evaluation is the drainage of the internal iliac veins (IILV). The incompetence of the main branches (b = branches) of the LIILV and RIILV drainage is assessed on the basis of the degree of their dilatation (usually above 5 mm), the direction of flow in the CDU (when feasible) and the dynamics of contrast enhancement in venographic examinations (CTV, MRV). The assessment of signal intensity changes in T2-weighted images, performed in the MRV examination can also become helpful.

#### 4.2.1. Grade I (GI)

**Figure 8:**
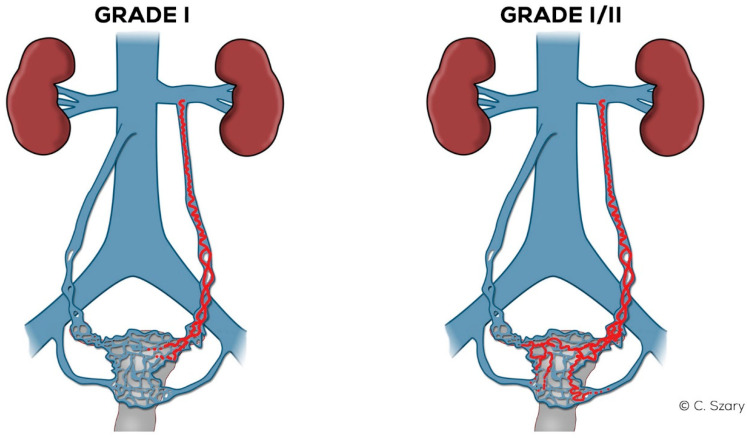
Schematic illustration of ovarian veins insufficiency: Grade I (GI) and Grade I/II (GI/II).

**Figure 9:**
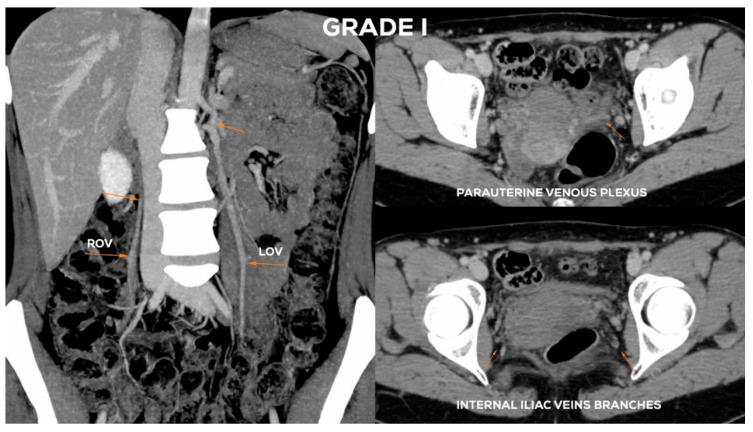
Typical findings in patient with Grade I ovarian veins insufficiency seen on a CTV images in coronal and axial planes.

In the case of GI (Figure 8) venous insufficiency, the LOV may demonstrate borderline competence in the supine examination. Only the upright or alternatively the sitting position of the patient and the performance of the Valsalva maneuver can reveal reflux, i.e., the dominance of reversed centrifugal flow. The LOV trunk usually shows a slight distension (most frequently Ø 5.5–6 mm). In GI incompetence the LOV ostium is usually not dilated. Dilation of para-uterine venous plexuses on the left side, if present, is slight (lPUV up to 5–5.5 mm). In the GI there is no overload of the left internal iliac vein outflow. The ROV trunk at the Grade I is non-dilated and fully efficient (Figure 9).

#### 4.2.2. Grade I/II (GI/II)

**Figure 10:**
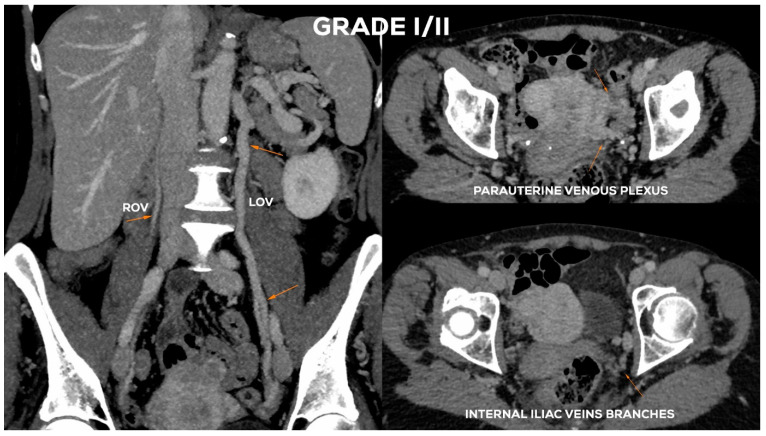
Typical findings in patient with Grade I/II ovarian veins insufficiency seen on a CTV images in coronal and axial planes.

In the Grade I/II (Figure 8), the LOV trunk is insufficient with relatively little dilatation (mean diameter about 6–6.5 mm). In the case of GI/II insufficiency, the LOV trunk ostium is usually slightly dilated. The distension of the venous plexuses is slight and affects the left side exclusively (lPUV up to 5.5 mm). In the GI/II, there is a mild volumetric overload of the LIILV drainage with bLIILV dilation to 5–6 mm. The right side does not show typical features of overload and insufficiency. Both the ROV trunk and its runoff do not show any signs of excessive distension. There is also no dilation of RIILV trunk and its branches (Figure 10).

#### 4.2.3. Grade II (GII)

**Figure 11:**
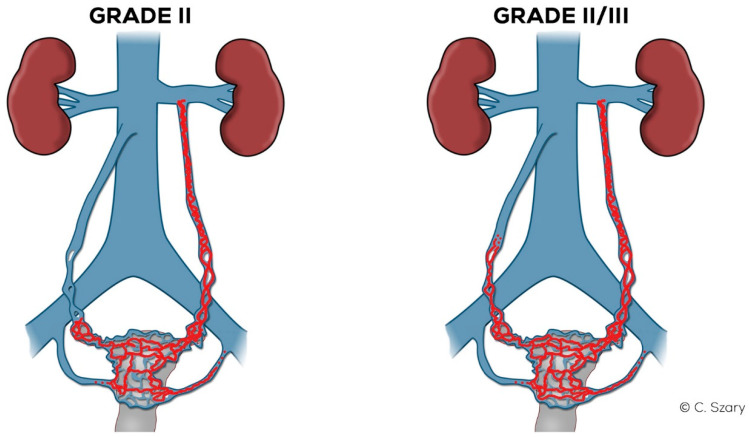
Schematic illustration of ovarian veins insufficiency: Grade II (GII) and Grade II/III (GII/III).

**Figure 12:**
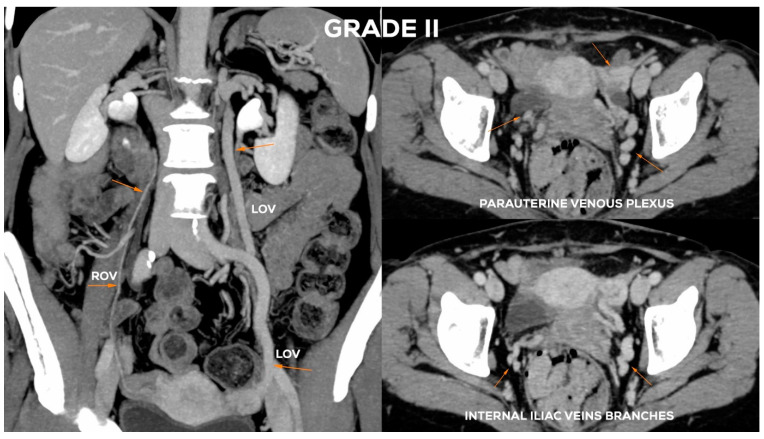
Typical findings in patient with Grade II ovarian veins insufficiency seen on a CTV images in coronal and axial planes.

In the Grade II (Figure 11), the LOV trunk is usually dilated to a greater extent (_Ø_LOV up to 7 mm). Ostium becomes distended (up to about 5–6 mm). In the GII distension of the venous plexuses of the left para-uterine region is significant, usually up to 6–6.5 mm. In GII insufficiency apart from distending in the LIILV runoff (bLIILV up to 6 mm), the right side is also slightly overloaded. The outflow of the ROV and, to a lesser extent, of the RIILV, gradually widen (rPUV up to 5.5 mm; bRIILV up to 5.5 mm). The ROV trunk remains efficient, despite moderate distension of its lower part, usually up to 5.5 mm (Figure 12).

#### 4.2.4. Grade II/III (GII/III)

**Figure 13:**
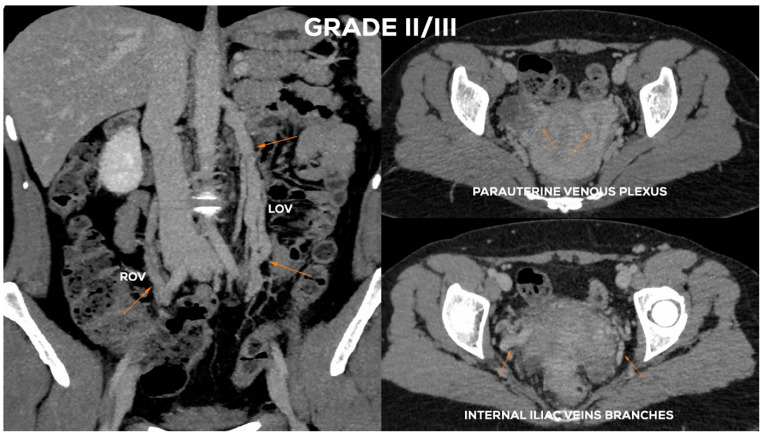
Typical findings in patient with Grade II/III ovarian veins insufficiency seen on CTV images in coronal and axial planes.

In the Grade II/III (Figure 11), the LOV trunk shows a clear insufficiency with dilatation up to 7.5–8 mm. Grade II/III distension of the left para-uterine venous plexuses is significant, usually up to 7 mm. The LIILV runoff is also dilated. Often the uterine vein or another branch of the LIILV (bLIILV) drainage becomes wider, even up to 6.5–7 mm. With GII/III insufficiency, there is a gradual volumetric overload of the right side with the distension of the lower ROV segment (_Ø_ROV up to 5.5–6 mm). The lower part of ROV when evaluated during the Valsalva maneuver or in a sitting position shows venous reflux. The middle and upper segment of the ROV is still efficient, although usually moderately widened. The venous plexuses of the right para-uterine region continue to distend (rPUV up to 6–6.5 mm). RIILV runoff (bRIILV up to 6 mm) is overloaded (Figure 13).

#### 4.2.5. Grade III (GIII)

**Figure 14:**
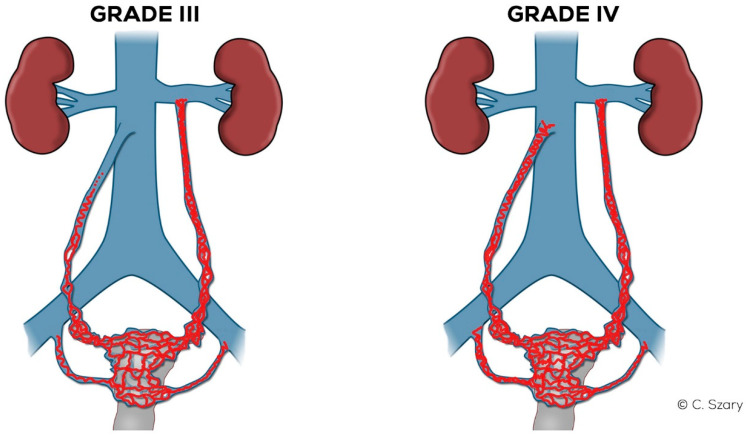
Schematic illustration of ovarian veins insufficiency: Grade III (GIII) and Grade IV (GIV).

The Grade III (Figure 14) usually shows a significant expansion of the LOV trunk (_Ø_LOV> 8 mm) with an evident distension of the ostial or sub-ostial segment. In the absence of valves, the diameter of the vessel can reach up to 10 mm. The widening of the venous plexuses of the left para-uterine region often reaches 7–8 mm. This leads to a significant overload of the uterine vein and distension of LIILV runoff (bLIILV usually widens to 7.5 mm).

**Figure 15:**
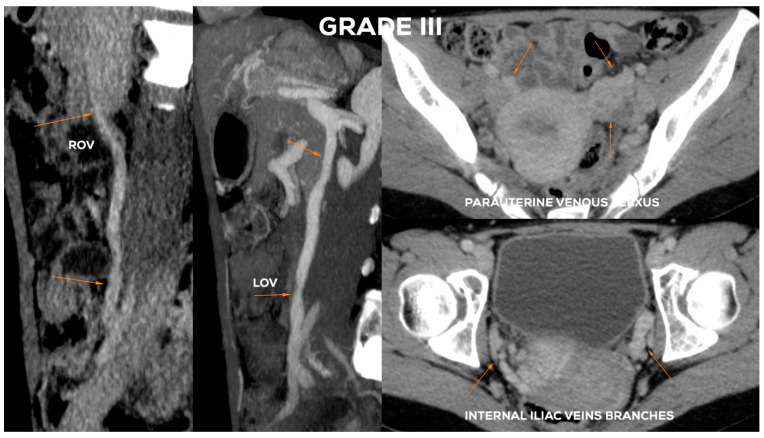
Typical findings in patient with Grade III ovarian veins insufficiency seen on CTV images in sagittal and axial planes.

In the GIII, the ROV trunk in the lower 2/3 usually reaches a diameter of 6.5–7.5 mm and is incompetent, with a relatively narrow upper segment, showing no obvious signs of insufficiency. GIII dilatation of the right para-uterine venous plexuses is usually evident (rPUV up to 6.5–7 mm). RIILV trunk and its branches (bRIILV up to 6.5 mm) are significantly overloaded (Figure 15).

#### 4.2.6. Grade IV (GIV)

**Figure 16:**
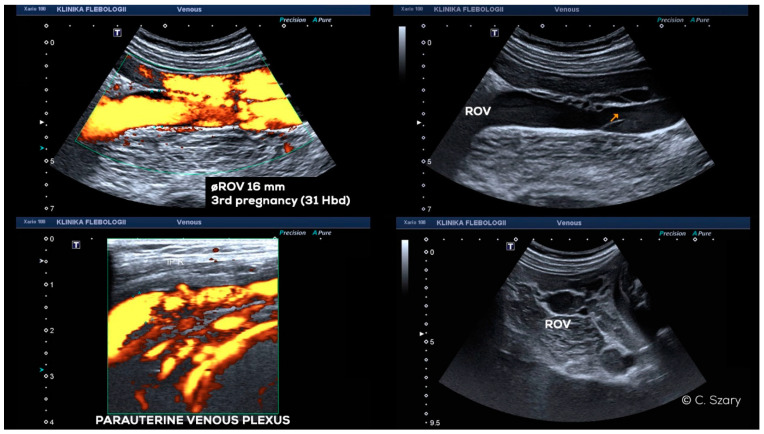
Transabdominal CDU findings of 35-year-old woman in third pregnancy (31 Hbd); distension of the ROV (16 mm) with reversed flow and dilated para-uterine venous vessels and varices of round ligament.

In the GIV (Figure 14), a significant distention of the LOV trunk is visible (often _Ø_LOV > 10 mm). This type of venous insufficiency advancement, in the absence of significant anatomical anomalies, is usually found in multiparous women giving birth more than three–four times. The degree of overload in the right ovarian axis in these women during pregnancy is so high (Figure 16) that after delivery a permanent enlargement occurs with the presence of bilateral para-uterine varices and usually a severe secondary overload of the internal iliac vein branches.

**Figure 17:**
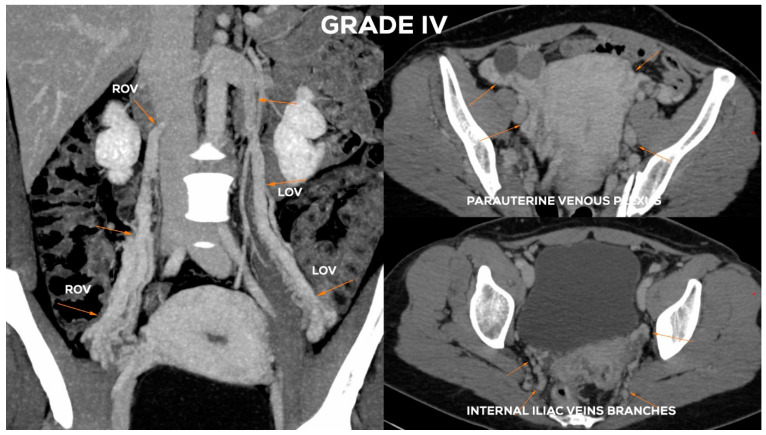
Typical findings in patient with Grade IV ovarian veins insufficiency seen on a CTV images in coronal and axial planes.

In the GIV, the ROV contrasts quite quickly with the discharge of a contrast agent from the IVC trunk through its dilated ostium (often Ø > 5–6 mm). In the case of full-axis insufficiency, the ROV often reaches a diameter of > 8 mm (Figure 17).

## 5. Discussion

Our research as well as the literature data show that the most important factors leading to the insufficiency of the ovarian vein axes, and consequently to the chronic insufficiency of the pelvic venous system, are the anatomical factors and the number of pregnancies.

**Figure 18:**
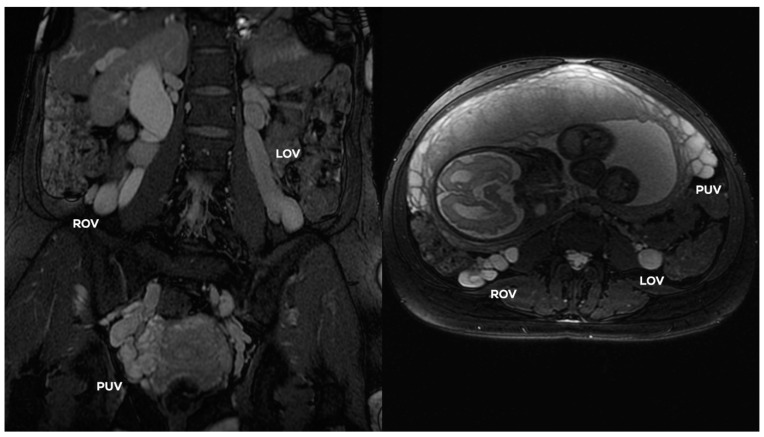
Prenatal MRI. Images in coronal and axial planes. Bilateral ovarian veins distention of 32-year-old woman in third pregnancy. (Courtesy of M. Bekiesinska-Figatowska, PhD; Department of Diagnostic Imaging, Institute of Mather and Child, Warsaw, Poland).

In addition to the anatomical predispositions discussed above, the number of pregnancies, in particular the number of full-term pregnancies, plays a key role in the development of venous insufficiency of the ovarian axes, and consequently chronic insufficiency of the pelvic veins. During each pregnancy, women experience a mechanical obstruction of blood outflow, resulting from the pressure of the enlarging uterus on the veins. This phenomenon is particularly evident after 26–28 weeks of pregnancy (Figure 18). The geometrical conformation and the width of female pelvis may play a role in PVI development in pregnancy. During ultrasound examinations in third trimester, we observe that slim women with a narrow pelvis more often have significant venous compression (IVC, LRV, LCILV) caused by gravid uterus with ovarian veins distension (Figure 18) [29].

An equally important mechanism is the hormone-dependent dilatation of the ovarian veins, the accompanying para-uterine venous plexuses, and the overload of the pelvic floor venous plexuses [30,31]. The distension of the ovarian venous bed is to compensate for the increase in blood volume capacity (by ca. 50–60%) during pregnancy [30] and to eliminate a significant acceleration of blood flow in the pelvic vessels (even 60 times greater than under normal conditions) [9]. Although the changes regress at least partially after delivery, subsequent pregnancies cause renewed overload to the ovarian axes which enlarges and sustains the degree of incompetence. The greater incidence of insufficiency of the ovarian veins on the left side compared to the right side suggests that in most cases the insufficiency of the ROV is due to an earlier (usually inter-gestational) reversal of blood flow in the LOV trunk, overload of the drained area and creation of a left-to-right vascular shunt mechanism.

As the phenomenon of ovarian venous insufficiency (LOV, ROV) is one of the most common forms of PVI, the ability to correctly assess this is of such great importance. The radiological evaluation of the runoff of the main ovarian axes, their influence on the circulation of the internal iliac veins and the collateral circulation generated in the abdominal cavity are the key factors in understanding venous hemodynamics and the phenomenon of PVI. The evaluation of the venous system should always be comprehensive, i.e., include the analysis of the veins in the abdominal cavity, the pelvis and the veins of the lower extremities and their mutual relations. The first examination in such an assessment should always be the CDU of the abdomino-pelvic venous system, beside lower limbs assessment.

Although many authors have emphasized difficulties in the assessment of abdominal and pelvic veins, either in DVT or PVI detection [25], according to our experience the thorough transabdominal CDU of the venous system, performed in sitting, standing and supine positions, in most cases enables the proper identification of patients with PVI. However, good experience in performing ultrasound examinations, as well as extensive knowledge of the anatomy and potential anatomical variations of venous system are required. Apart from the experience and diagnostic skills of the examiner, good-quality equipment and appropriate preparation of the patient for the examination are further issues which influence the quality of TAU exam. When fulfilling all above mentioned conditions, TAU may be of help in the assessment of PVI, especially in the imaging of ovarian veins and para-uterine venous plexuses [16,18,19]. When compared to CTV, the sensitivity and specificity of CDU in the detection of LOV dilatation is quite high (83–100%), whereas in case of dilated ROV it is slightly worse (67%) [19].

In order to precisely determine the degree of ovarian veins insufficiency, the results of the CDU examination are verified either in CTV or MRV. Apart from the morphology, the dynamics of the contrast enhancement, and a residual contrast agent retention in the venous system are assessed. Depending on the type of pathology, or clinical situation, other imaging modalities such as IVUS or descending phlebography may be used. However, according to our experience, in most cases CTV and MRV techniques are sufficient. The latter is preferred in patients of reproductive age, and with the suspicion of other pathologies in the pelvis, e.g., endometriosis, adenomyosis, or adhesions after surgical interventions.

When determining the final diagnosis, it should be considered that most abdominal and pelvic venous examinations are performed in the supine position. Therefore, when performing ultrasound and phlebographic examinations (at the pre- and/or intraoperative assessment stage), it may be helpful to position the patient’s body in a tilt.

**Figure 19:**
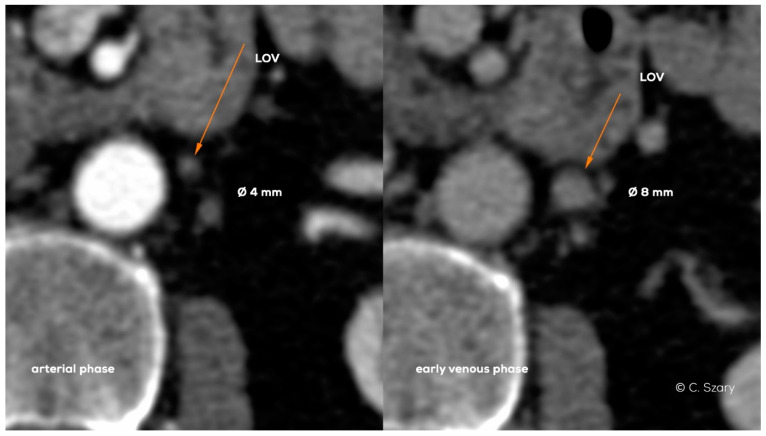
Axial CT images in arterial and early venous phase show significant difference in left ovarian vein (LOV) diameters.

The important element for patients’ preparation is their adequate hydration, both due to the contrast agent administration and the proper filling of the vascular bed. Another equally important factor influencing the interpretation of imaging is its duration, usually several minutes for typical CTV vs. 45–50 min for MRV examination. Apart from test duration, the contrast phase and respiratory phases also significantly affect the accuracy of vein diameter measurements, which translates into the final result of evaluation (Figure 19).

It is obvious that the accuracy of this assessment will affect the precision with which both the diagnosis and the proper causes (as well as the mechanism) of the development of venous disease are determined. This translates directly into the options and safety of treatment, which today is mainly carried out with the use of minimally invasive, endo-venous techniques. For this reason, the improvement of communication between diagnosticians and treatment specialists, including standardizing the method of radiological assessment and introducing an appropriately simple and transparent scheme of grading the efficiency of the ovarian vein axes into common clinical practice, seems to be helpful in many cases when deciding on the course of treatment of patients with venous insufficiency.

Our own experience with this classification has led to a specific therapeutic approach, as follows. Patients qualified into groups I/II to II/III (Table 4) usually undergo the removal of reflux in the left ovarian axis or in the ovarian-iliac axis, while those qualified into groups III and IV require bilateral treatment. The embolization of both ovarian axes along with their drainage, or of individual inefficient branches of the internal iliac veins, is thus performed in these advanced cases.

The impact of combined diagnostic imaging and the key role of CDU assessment in the planning of treatment for patients with PVI, were discussed in a recent consensus document [25]. When addressing the treatment of the varicose veins of the lower limbs, the preliminary PVI treatment has been considered controversial. However, our experience, in agreement with other authors [32], suggests that PVI treatment as first step may represent a primary indication in selected cases. This approach may complement and impact the outcome of the subsequent treatment in the lower limbs.

## 6. Conclusions

The proposed hemodynamic and morphological classification of ovarian venous insufficiency is a tool which may facilitate communication among specialists dealing with the diagnosis and treatment of venous insufficiency. A clear classification scheme also helps to understand the mechanisms responsible for the occurrence of PVI, including its most common form, i.e., ovarian veins insufficiency, which is the key to the proper treatment of patients with chronic venous insufficiency. Commonly available diagnostic techniques, especially CDU as well as MRV and CTV examinations, in the vast majority of cases are sufficient to achieve a proper diagnosis and to plan an appropriate treatment. The results of the latest research, as well as the authors’ own experience, suggest that this approach may give long-term, positive results in the treatment of chronic venous insufficiency, with a possibly lower degree of varicose vein recurrence [32,33,34].

## Figures and Tables

**Figure 4:**
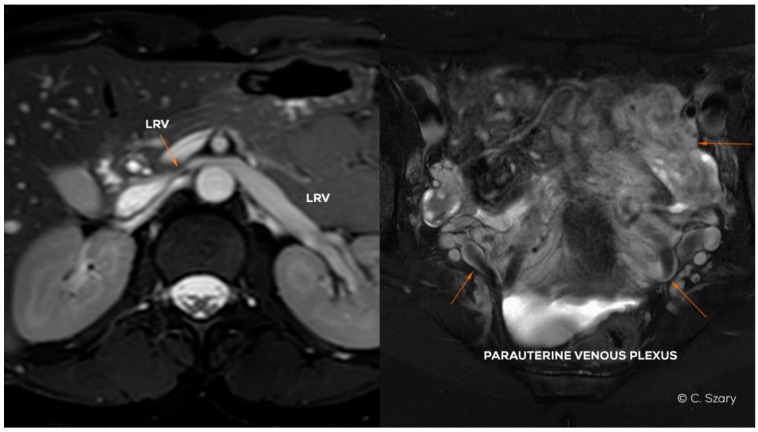
Magnetic resonance (MR) images (BTFE and T2-weighted sequences in axial plane) presenting LRV partial hypoplasia and secondary para-uterine varices caused by LOV insufficiency.

**Figure 5:**
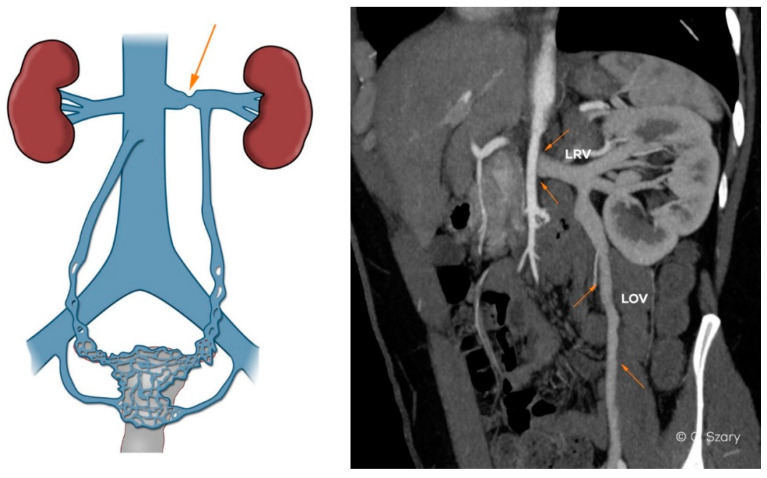
Typical anatomy of nutcracker syndrome. Insufficiency of LOV on computed tomographic venography (CTV) image.

**Table 1:** Characteristics of assessed group. The values shown in table represent mean ± SD, or the number (%) of patients reporting evaluated symptom (or feature) in the group, respectively.

Parameter or Variable	Value (*n* = 535)
**Age**	42.3 ± 10.5
**Mean number of pregnancies (P)** P0 P1 P2 P3 P4+	1.7 ± 1.4 132 (24.7%) 104 (19.4%) 176 (32.9%) 78 (14.6%) 45 (8.4%)
**Mean number of deliveries**	1.5 ±1.2
**Predominant signs and symptoms:** dilated reticular and/or “spider” veins lower limbs pain/discomfort abdomino-pelvic pain/discomfort	197 (33.5%) 252 (47.1%) 104 (19.4%)
**Clinical CVD classification (CEAP):** C1 C2 C3 C4	56 (10.5%) 314 (58.7%) 128 (23.9%) 37 (6.9%)

**Table 2:** The most important anatomical factors correlated to LOV insufficiency

- absence, hypoplasia or postthrombotic septations of suprarenal or hepatic segment of IVC
- absence or (partial) hypoplasia of LRV (Figure 4)
- LRV extrinsic entrapment syndrome (“nutcracker phenomenon”) (Figure 5)
- postthrombotic septs or intraluminal webs, channels and spurs in LRV
- extrinsic compression of retroaortic LRV
- ascending course of the LRV (hilar segment below its distal part)
- distended ostium of the LOV draining into the LRV
- lack of an ostial or subostial valve in the LOV trunk
- atypical LOV drainage (the most frequent form draining into the main branch of LRV)
- combined anatomy of the LOV system (e.g., duplication of the main trunk)

**Table 3:** The most important anatomical factors correlated to ROV insufficiency.

- absence, hypoplasia or postthrombotic septations of suprarenal or hepatic segment of IVC
- absence or hypoplasia of RRV
- atypical ROV drainage (draining into the main branch of the RRV or accessory RRV)
- high localized ostium of ROV to IVC (on its ventral side)
- distended ostium of the ROV typically draining into the IVC
- lack of an ostial or subostial valve in the ROV trunk
- combined anatomy of the ROV system (e.g., duplication of the ROV trunk)

**Table 4:** Grades of ovarian veins insufficiency in pelvic venous incompetence. Acronyms: _Ø_LOV/_Ø_ROV–diameter of left or right ovarian vein; incLOV/incROV–incompetence of left or right ovarian vein; lPUV/rPUV–left or right para-uterine veins; bLIILV/bRIILV–branches of left or right internal veins. All numeric values are in “mm”.

	øLOV	incLOV	lPUV	bLIILV	øROV	incROV	rPUV	bRIILV
**GI**	<6	(−/+)	<5	<5	<5	(−)	<5	<5
**GI/II**	6–6.5	(+)	<5.5	<5.5	<5	(−)	<5	<5
**GII**	<7	(+)	<6.5	<6	<5.5	(−)	<5.5	<5.5
**GII/III**	7.5–8	(++)	<7	<7	<6	(−/+)	<6.5	<6
**GIII**	>8	(++)	7–8	<7.5	<7.5	(+/++)	<7	<6.5
**GIV**	>10	(+++)	>8	>8	>8	(+++)	>7.5	>7

## Data Availability

Not applicable.
